# Protective effects of manganese(II) chloride on hyaluronan degradation by oxidative system ascorbate *plus* cupric chloride

**DOI:** 10.2478/v10102-010-0001-7

**Published:** 2010-03-29

**Authors:** Katarína Valachová, Grigorij Kogan, Peter Gemeiner, Ladislav Šoltés

**Affiliations:** 1 Institute of Experimental Pharmacology & Toxicology, Slovak Academy of Sciences, SK-84104 Bratislava, Slovakia; 2 Institute of Chemistry, Center for Glycomics, Slovak Academy of Sciences, SK-84538 Bratislava, Slovakia; 3 Directorate Health, Directorate General Research, European Commission, B-1049, Brussels, Belgium

**Keywords:** hyaluronan, Weissberger oxidative system, reactive oxygen species, dynamic viscosity, ascorbic acid, Fenton reaction, manganese(II) chloride

## Abstract

The degradation of several high-molar-mass hyaluronan samples was investigated in the presence of ascorbic acid itself and further by an oxidative system composed of ascorbic acid plus transition metal ions, i.e. Fe(II) or Cu(II) ions. The latter oxidative system imitates conditions in a joint synovial fluid during early phase of acute joint inflammation and can be used as a model for monitoring oxidative degradation of hyaluronan under pathophysiological conditions. The system Cu(II) *plus* ascorbate (the Weissberger oxidative system) resulted in a more significant decrease of hyaluronan molar mass compared to the oxidative system Fe(II) plus ascorbate. Addition of manganese(II) chloride was found to decrease the rate of the oxidative damage of hyaluronan initiated by ascorbate itself and by the Weissberger system.

## Introduction

Some biogenic transition metals, such as iron, copper, manganese, zinc, and cobalt, participate in the control of various metabolic and signaling pathways. However, their versatile coordination chemistry and redox properties allow them to escape the control mechanisms, such as homeostasis, transport, compartmentalization, and binding to the designated tissue and cell constituents (Valko *et al*., 2005; Šoltés and Kogan, 2009).

In a wide variety of *in vitro* systems, Fe(II) salts and/or non-enzyme complexed ferrous cations (e.g. Fe(II)-EDTA) were shown to enhance oxygen radical damage by increasing the production of an oxidative species generally believed to be the hydroxyl free radical. Iron ions are known to cause peroxidation of (polyunsaturated) fatty acids in lipids (LH) and generate peroxyl lipid radicals (LOO·) by a sequence of the following reactions

(1) $$\text{2Fe(II)+2}{\text{O}}_{\text{2}}\text{↔ 2Fe(III)+2}{\text{O}}_{\text{2}}^{\mathrm{\cdot -}}\text{(redox reaction; reversible)}$$

(2) $$\text{2}{\text{O}}_{\text{2}}^{\mathrm{\cdot -}}\text{+ 2}{\text{H}}^{\text{+}}{\text{→H}}_{\text{2}}{\text{O}}_{\text{2}}\text{+}{\text{O}}_{\text{2}}\text{(dismutation reaction)}$$

$$\text{2Fe(II)+}{\text{O}}_{\text{2}}\text{+ 2}{\text{H}}^{\text{+}}\text{→2Fe(III)+}{\text{H}}_{\text{2}}{\text{O}}_{\text{2}}\text{ net reaction}$$

(3) $$\text{Fe(II)+}{\text{H}}_{\text{2}}{\text{O}}_{\text{2}}\text{→Fe(II)+}\cdot \text{OH + H}{\text{O}}_{}^{-}\text{(Fenton reaction)}$$

(4) $${\text{O}}_{2}^{\mathrm{\cdot -}}\text{+}{\text{H}}_{\text{2}}{\text{O}}_{\text{2}}\text{↔H}{\text{O}}^{\cdot }\text{+ H}{\text{O}}^{-}\text{+}{\text{O}}_{\text{2}}\text{(Haber-Weiss reaction; reversible)}$$

(5) $$\text{LH}{+}^{\cdot }\text{OH→}{\text{L}}^{\cdot }\text{+}{\text{H}}_{\text{2}}\text{O}$$

(6) $${\text{L}}^{\cdot }\text{+}{\text{O}}_{\text{2}}\text{→LO}{\text{O}}^{\cdot }$$

The LOO· radicals propagate the lipid peroxidation chain reactions LOO + LH → LOOH + L·, and LOOH oxidizes ferrous ions yielding alkoxyl lipid radicals LOOH + Fe(II) → LO· + Fe(III) + HO^–^, whereas the generated LO· radicals participate in the propagation phase of the lipid peroxidation reaction LO· + LH → LOH + L·.

Ascorbate (Asc^–^) belongs to the most efficient (bio)reductants capable to keep iron ions in the lower oxidation state and/or to recycle Fe(III) to Fe(II). The so-called iron-catalyzed ascorbate auto-oxidation yields an intermediate – the semidehydroascorbate radical (Asc·^−^) – a low-reactive radical that can undergo dismutation/disproportionation reaction to form Asc^–^ and dehydroascorbate (DHA)

(7) $$\text{As}{\text{c}}^{-}\text{+ Fe(III)→As}{\text{c}}^{\mathrm{\cdot -}}\text{+ Fe(II)}$$

(8) $$\text{2As}{\text{c}}^{\mathrm{\cdot -}}\text{→As}{\text{c}}^{-}\text{+ DHA}$$

Alternatively, complexes of Fe(II) ions and dioxygen are also assumed to yield reactive species of unknown nature, which are subsequently able to oxidize biological material (Qian and Buettner, 1999; Flemmig and Arnhold, 2007).

**Scheme 1:**
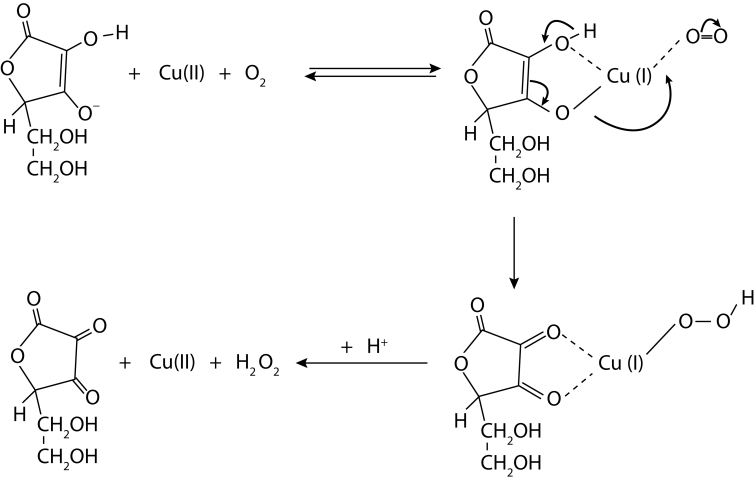
Generation of H_2_O_2_ by Weissberger's system from ascorbate and Cu(II) under aerobic conditions (adapted from Fisher and Naughton, 2005).

A combination of ascorbate *plus* Cu(II) under aerobic conditions, the so-called Weissberger's system (Weissberger *et al*., 1943; Khan and Martell, 1967), gives rise directly to hydrogen peroxide (cf. Scheme 1, Fisher and Naughton, 2003; 2004; 2005) and, taking into account the fact that ascorbate reduces Cu(II) to cuprous ions, it is feasible to assume that at copper-catalyzed ascorbate auto-oxidation, ·OH radicals should be generated by a Fenton-type reaction Cu(I) + H_2_O_2_ → Cu(II) + ·OH + HO^–^ . This conclusion was recently supported by an unambiguous proof of production of hydroxyl radicals in a system comprising ascorbate *plus* CuCl_2_ by using EPR spin-trap technique, applying such spin traps as 5,5-dimethyl-1-pyrroline-*N*-oxide (DMPO) and 5-(diisopropoxyphosphoryl)-5-methyl-1-pyrroline-*N*-oxide (DIPPMPO) (Šoltés *et al*., 2006).

The oxidative damage of various biomolecules (lipids, enzymes, DNAs, *etc*.) with (catalytic) participation of inorganic Fe and/or Cu salts/complexes has been clearly demonstrated in many *in vitro* assays. Yet under physiological conditions, taking into account the negligible availability of “free catalytic iron”, the significance of e.g. the Fenton reaction can not be fully clarified. The average-weight human body contains approximately 4–5 g iron bound to hemoglobin, myoglobin, cytochromes, iron-containing enzymes, and to the iron storage proteins – ferritin, transferrin, and hemosiderin. Similarly, about 95% of copper circulating in the blood is bound to ceruloplasmin. Further copper is bound/ligated to albumin, transcuprein, and CuZn-superoxide dismutase.

Unlike Fe and Cu, inorganic salts/complexes of the biogenic transition metal – Mn – are known to occur at high concentrations in certain cells. As reported, manganese concentrations in most adult human tissues range between 3 and 20 μM (Roth, 2006). The results of several *in vitro* studies imply that Mn in various forms does indeed inhibit damage mediated by ·OH radicals, however only if their production is dependent on the presence of O_2_ ·^–^ or H_2_O_2_ . Thus, Mn complexes appear to interact with ·OH, as well as with O_2_ ·^–^ or H_2_O_2_ , in a fundamentally different fashion than those of Fe and Cu (Cheton and Archibald, 1988).

**Figure 1:**
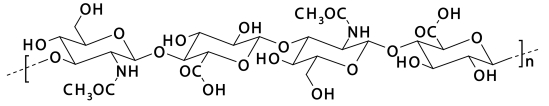
Hyaluronan – the acid form.

Hyaluronan (HA; Figure 1) is a high-molar-mass glycosaminoglycan that has important functions in living organisms (Kogan *et al*., 2007; 2008). HA macromolecules with molar mass equaling several MDa are extruded into synovial fluid (SF) by synoviocytes/hyalocytes – the cells embedded in the synovial membrane. Free/non-associated HA present in SF determines its unique viscoelastic properties required for maintaining the proper functioning of the joints in vertebrates.

It is noteworthy that the half-life of HA in SF is only a few hours. Although the relatively fast HA catabolism is controlled by hyaluronidases in most tissues, in SF – due to the absence of these enzymes – different mechanisms are implemented in the rapid hyaluronan catabolism. One of the possible alternative mechanisms involved in the joints of healthy individuals is the oxidative/degradative action of reactive oxygen species (ROS) generated during the ascorbate auto-oxidation catalyzed by the transition metal (Fe and/or Cu) ions (Šoltés *et al*., 2005). Evidence exists that ROS are responsible for HA degradation in inflammatory joint diseases, such as osteoarthritis and rheumatoid arthritis (RA). HA involvement in activation and modulation of the inflammatory response includes also its scavenging action toward ROS, such as ·OH radicals (Mendoza *et al*., 2007).

As demonstrated in many studies, under aerobic conditions a ternary system, comprising HA macromolecules *plus* ascorbate and traces of iron or copper ions, induces gradual decrease of the viscosity of the HA solution, as a result of fragmentation/degradation of HA macromolecules. However as to the effects of manganese on HA degradation invoked by ascorbate auto-oxidation, not a single study has been so far published. The impact of Mn(II) ions is stipulated by a known catalytic participation of this essential transition metal in the function of hyaluronan synthase(s) (Johnson *et al*., 2001; Weigel and DeAngelis, 2007).

As reported, certain Mn(II) complexes, including biologically relevant Mn(II) pyrophosphate and Mn(II) polyphosphate, can act as very effective antioxidants by indirectly suppressing or blocking ·OH formation due to Fenton-, Haber-Weiss-, xanthinoxidase-Fe-EDTA-, or Fe(III)-H_2_O_2_- type reactions, precisely as do superoxide dismutase and catalase (Cheton and Archibald, 1988). It has been established that these two major antioxidatively acting enzymes are barely detectable in rheumatoid synovial fluid (Wong *et al*., 1981; Parsons *et al*., 2002). Their levels in SF, if any, do not exceed 1μg/mL and 50 ng/mL, respectively (McCord, 1974).

Potential use of manganous salts/complexes for protecting lipids against oxidative stress has been demonstrated in several *in vitro* and *in vivo* studies (Cheton and Archibald, 1988; Shukla and Chandra, 1981; Archibald and Fridovich, 1982; Cavallini *et al*., 1984; Chang and Kosman, 1989; Varani *et al*., 1991; Coassin *et al*., 1992; Gray and Carmichael, 1992; Singh *et al*., 1992; Tampo and Yonaha, 1992; Sakurai *et al*., 1997; Sziraki *et al*., 1998; 1999; Hussain and Ali, 1999; Worley *et al*., 2002). The results reported indicate that Mn(II) scavenges superoxide anion radicals already at nanomolar concentrations, whereas its micromolar concentrations are required to scavenge hydroxyl radicals. Increasing concentrations of manganese suppress lipid peroxidation even more strongly, and complete inhibition is reached at concentrations of 30 μM Mn(II) (Shukla and Chandra, 1981).

Manganese may act as a chain-breaker in inhibiting iron-induced lipid peroxidation chain reactions (Sziraki *et al*., 1998; 1999), and as proposed (Coassin *et al*., 1992; Sziraki *et al*., 1998), Mn(II) may scavenge peroxyl lipid radicals *via* the following reaction

(9) $$\text{LO}{\text{O}}^{\cdot }\text{+ Mn(II)+}{\text{H}}^{+}\text{→LOOH + Mn(III)}$$

quenching in this way the propagation reactions of lipid peroxidation caused by hydroxyl radicals generated by pro-oxidative transition metals such as iron and copper.

The aim of the present study was to investigate the function of trace concentrations of Fe(II), Cu(II), as well as of Mn(II) in ascorbate auto-oxidation, in which hyaluronans of various molar masses are involved as indicators of pro- or antioxidative properties of the system.

## Material and methods

### Biopolymers

Six hyaluronan samples, covering by their molar-mass averages (M_w_) the range of 0.43 to 1.3 MDa (cf. Table 1), were kindly donated by or purchased from the following HA manufacturers: Genzyme Corporation, Cambridge, MA, U.S.A; Sigma Chemicals Co., St. Louis, MO, U.S.A.; Lifecore Biomedical Inc., Chaska, MN, U.S.A.; and CPN Ltd., Ústí nad Orlicí, Czech Republic (Stankovská *et al*., 2004, Šoltés *et al*., 2007). In the HA samples P9706-6 and P9710-2, the contents of the following (transition) metals were stated by manufacturer (in ppm): Fe = 27 and 13; Pb = 6 and 7; Cu = 3 and 4, respectively; Cr, Co, Ni < 3 and As, Cd, Hg < 1, in both samples [“Certificate of Analysis” (Lifecore Biomedical Inc., Chaska, MN, U.S.A.)].

**Table 1:** Summary of the characteristic parameters of the six HA samples.

Sample designation	Parameter^a^[unit]		
	
	**M**_**w**_[kDa]	**M**_**w**_**/M**_**n**_[−/−]	**Rg** [nm]
**B22157**	1340	1.50	129.8
**53H0439**	1017	1.82	130.7
**P9710-2A^b^**	808.7	1.63	110.0
**P9706-6**	803.9	1.64	107.9
**CPN**	659.4	1.88	97.4
**1-9100-1**	426.2	1.84	77.2

^a^M_w_ − weight-average of molar masses, M_w_/M_n_ − polydispersity index, and Rg − z-average of the radius of gyration.

^b^Aged HA sample (Šoltés *et al*., 2007).

### Chemicals

Analytical purity grade NaCl and CuCl_2_·2H_2_O were from Slavus Ltd., Bratislava, Slovakia; FeCl_2_·4H_2_O was purchased from Penta CZ Ltd., Chrudim, Czech Republic; MnCl_2_·4H_2_O was from Lachema CZ Ltd., Brno, Czech Republic; ascorbic acid (AA) was from Merck KGaA, Darmstadt, Germany. Redistilled de-ionized quality grade water with ≤ 0.055 μS/cm conductivity was prepared by using the TKA water purification system (Water Purification Systems GmbH, Niederelbert, Germany).

### Hyaluronan degradation/Rotational viscometry

The HA sample (20 mg) was dissolved overnight in the dark in 0.15 M NaCl in two steps: First, 4.0 mL of the solvent was added in the morning. The next 3.95 mL of the solvent was added after 6 h. On the following morning, 50.0 µL of 16.0 mM AA dissolved in 0.15 M NaCl was added to the HA solution and blended for 30 s. The resulting solution (8 mL) containing HA (2.5 mg/mL) and AA (100 µM) was transferred into the Teflon® cup reservoir of the Brookfield LVDV-II+PRO rotational viscometer (Brookfield Engineering Labs., Inc., Middleboro, MA, U.S.A.). The recording of the viscometer output parameters started 2 min after the experiment onset. The solution dynamic viscosity (η) was measured at 25.0±0.1 °C in 3 min intervals for up to 5 h. The viscometer Teflon® spindle rotated at 180 rpm, i.e. at the shear rate equaling 237.6 s^−1^ (Šoltés *et al*., 2007).

When the effect of the addition of a single biogenic transition metal was investigated, the second portion of the aqueous NaCl solvent was only 3.90 mL. On the following morning, the addition of 50.0 µL of 16.0 mM AA to the HA solution was followed by the admixing of 50.0 µL of appropriate FeCl_2_, CuCl_2_, or MnCl_2_ solutions in 0.15 M aqueous NaCl. The concentration of the biogenic transition metal salt in the system was 0.5 or 5.0 µM when using FeCl_2_; 0.1, 1.0, or 5.0 µM with CuCl_2_; and 0.5 µM with MnCl_2_.

When the (inhibitory) action of the Mn(II) ions on HA degradation by the system comprising AA (100 µM) and CuCl_2_ (1.0 µM) was assessed, the second portion of aqueous NaCl was 3.85 mL. Fifty µL of the MnCl_2_ solution in 0.15 M aqueous NaCl was added to adjust the final Mn(II) concentration to 30 µM. Three different application schemes of AA and of the two metal ions were tested: (**i**) Mn(II) followed by (AA) and Cu(II); (**ii**) AA followed by Mn(II) and Cu(II); and (**iii**) Mn(II) followed by Cu(II) and AA.

In each case, a homogenous solution was obtained after 30 s of moderate stirring of the mixture upon addition of AA or transition metals.

Under the above specified experimental settings, the torque values were in the interval between 82 and 23 %.

## Results

**Figure 2:**
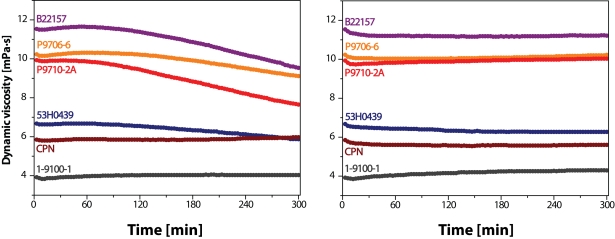
Time dependence of dynamic viscosity of HA solutions. Left panel: Solutions of hyaluronan samples with addition of 100 µM AA. Right panel: Solutions of hyaluronan samples with addition of 100 µM AA and 0.5 µM MnCl_2_.

As shown in Figure 2, left panel, the dynamic viscosity *vs*. time relationship of the solutions containing HA (2.5 mg/mL) and AA (100 μM), with the exception of the two samples, namely CPN and 1-9100-1, indicates the presence of two distinct regions: (***i***) rheopectic and (***ii***) the region that should be assigned to the degradation of HA macromolecules. As calculated from the Certificate of Analysis of the HA samples P9706-6 and P9710-2, solutions of these two samples contained the following concentrations of iron and copper ions: 1.209 and 0.118 µM (P9706-6) or 0.582 and 0.157 µM (P9710-2), respectively. Due to the presence of these biogenic transition metal ions, the Fe(III)/Fe(II) and Cu(II)/Cu(I) catalyzed ascorbate auto-oxidation leads to the generation of ·OH radicals that, after a certain initiation period, promote degradation of HA macromolecules, which is manifested by a gradual decrease of the solution dynamic viscosity.

The results presented in Figure 2, right panel, indicate that even a submicromolar addition of Mn(II) ions (0.5 µM) prolong the rheopectic region in the η *vs*. time plot up to 300 min, the total time of monitoring, which is especially recognizable in the case of the samples B22157, P9706-6, and P9710-2A. No or only small changes occurred using 0.5 µM Mn(II) ions with HA samples having lower molar mass. It should be noted that most curves implied that a slight decrease in viscosity took place after addition of Mn(II).

**Figure 3:**
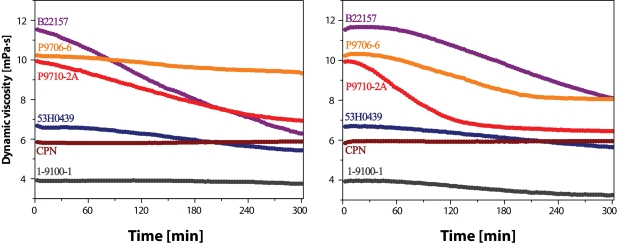
Time dependence of dynamic viscosity of HA solutions. Left panel: Solutions of hyaluronan samples with addition of 100 µM AA and 0.5 µM FeCl_2_. Right panel: Solutions of hyaluronan samples with addition of 100 µM AA and 0.1 µM CuCl_2_.

Contrary to an “antioxidative” action of MnCl_2_, an identical 0.5 µM concentration of FeCl_2_, or even smaller 0.1 µM concentration of CuCl_2_ had a “pro-oxidative” effect on the degradation of HA macromolecules in most samples (compare the data presented in left and right panels in Figure 3 with those in Figure 2, left panel). The only exception was observed with the sample CPN, in which, however, a relatively high content of “a contaminant” – transition metal Mn ions – was detected (Prof. A. Staško, Slovak Technical University, Bratislava, Slovakia, personal communication).

**Figure 4:**
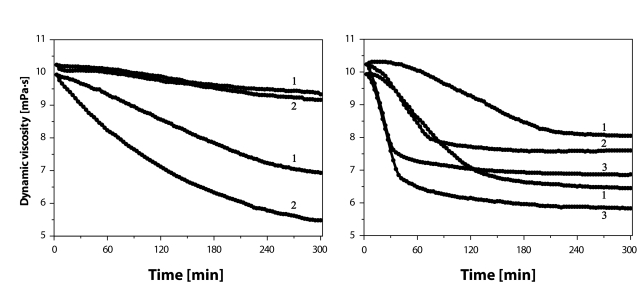
Time dependence of dynamic viscosity of HA P9710-2A (black lines) and P9706-6 (grey lines) sample solutions. Left panel: Solutions of hyaluronan samples with addition of 100 µM AA and of 0.5 (**1**) or 5.0 µM (**2**) FeCl_2_. Right panel: Solutions of hyaluronan samples with addition of 100 µM AA and of 0.1 (**1**), 1.0 (**2**) or 5.0 µM (**3**) CuCl_2_.

The pro-oxidative effect of the addition of Fe or Cu ions is clearly indicated in a concentration-dependent manner (cf. Figure 4, left and right panels). A slight difference should be however pointed out concerning the “nominal” η_2′_ values, i.e. the values observed at the 2^nd^ min after addition of metal ions in different concentrations (0.1–5.0 µM) to the solutions containing HA (2.5 mg/mL) and AA (100 µM). For a better visualization, we shifted the η_2′_ value of the solutions containing metal salts to the value η_2′_ valid for the solutions comprising only HA and AA. By such “normalization”, the changes in the values of dynamic viscosity caused by different amounts of added FeCl_2_ and CuCl_2_ became more visible (as presented in both panels of Figure 4). As can be seen, the character of the time dependence of η value upon the addition of FeCl_2_ can be described as a gradual monotonous concentration-dependent decline, while the addition of CuCl_2_ resulted in a literally “dramatic” drop of η value in a very short time interval, after which its decrease continued, however at a much lower rate. A possible explanation of this dissimilarity may lie most probably in different reaction kinetics of the processes leading to generation of ROS in a system ascorbate *plus* FeCl_2_ and in that comprising ascorbate *plus* CuCl_2_.

**Figure 5:**
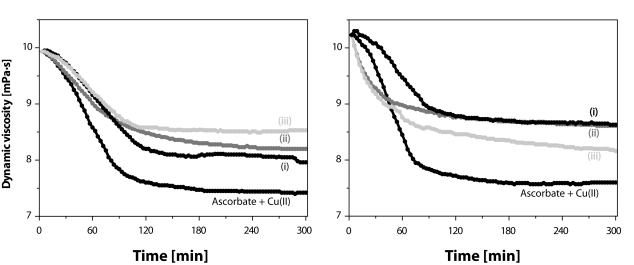
Time dependence of dynamic viscosity of HA sample solutions P9710-2A (left panel) and P9706-6 (right panel) after addition of AA and CuCl_2_; (**i**) MnCl_2_ followed by AA and CuCl_2_; (**ii**) AA followed by MnCl_2_ and CuCl_2_; and (**iii**) MnCl_2_ followed by CuCl_2_ and AA. The concentrations used were 100 µM AA, 1.0 µM CuCl_2_, and 30 µM MnCl_2_.

The results presented in Figure 5 illustrate the effect of Mn(II) addition on η *vs*. time dependence of the solutions comprising high-molar-mass hyaluronan (2.5 mg/mL), ascorbate (100 µM), and CuCl_2_ (1.0 µM). The addition of MnCl_2_ in a relatively high concentration (30 µM) resulted in a significant decrease of the degradation of HA macromolecules, however, none of the used application schemes (**i**, **ii**, **iii**) resulted in total inhibition of the biopolymer degradation. While in the case of sample P9710-2A using the application scheme (**iii**), the sample damage was decreased to ca. 44% (cf. Figure 5, left panel), in the case of sample P9706-6, scheme (**i**) proved to be the most efficient, i.e. extent of degradation decreased to ca. 39% (cf. Figure 5, right panel).

## Discussion

Under physiological conditions as well as at the early stage of acute-phase joint inflammation, contribution of ascorbate auto-oxidation to the non-enzymatic catabolism of high-molar-mass hyaluronan in SF is plausible since: (***a***) in a healthy human being, the content of free HA macromolecules in SF is 1.4–3.6 mg/mL (Kogan *et al*., 2007); (***b***) the concentration of ascorbate in SF of healthy subjects reaches the values closely to those established in blood serum, i.e. 40–140 μM (Wong *et al*., 1981); (***c***) the total concentrations of Fe and Cu ions in SF of healthy human equal 5.2 and 4.3 μM, respectively, and they rise under pathological/inflammatory conditions such as osteoarthritis (OA) and RA; (***d***) in SF of the individuals suffering from RA, the total Cu concentration is increased more than three-fold compared to that of the healthy population (Niedermeier and Griggs, 1971) and their Cu concentration in SF ultrafiltrate equals 0.125±0.095 μM (Naughton *et al*., 1995); (***e***) at ascorbate auto-oxidation with the catalytic contribution of Cu(II) traces, direct conversion/transformation of O_2_ to H_2_O_2_ takes place (Weissberger *et al*., 1943; Khan and Martell, 1967; Fisher and Naughton 2003; 2004; 2005); (***f***) the average concentration of Mn ions in SF of healthy persons and patients with RA is relatively low (ca. 0.42 and 0.44 μM, respectively) (Niedermeier and Griggs, 1971).

Investigation of the participation of biogenic transition metals in oxidative damage of high-molar-mass HA appears to be very simple. However, at least two main technical/experimental obstacles should be pointed out. First: due to an extremely high aggressiveness of the oxidative/radical processes, the materials, which come into contact with e.g. the ·OH radicals, should be non-metallic (preferably made of glass or Teflon^®^ (Stankovská *et al*., 2004; 2005). Second: at present, such studies are substantially limited due to the unavailability of the ultrapure HA reference preparations with sufficiently high molar masses completely devoid of contaminating metals.

The above limitations have been circumvented in our studies by using the Brookfield LVDV-II+PRO rotational viscometer equipped with a cup-reservoir and spindle both home-made of Teflon^®^, and by using HA samples which purity was sufficiently high. The samples coded P9706-6 and P9710-2 with the known content of the given (transition) metals allowed us to calculate the concentrations of iron and copper ions in the solutions of these samples. The other four samples, despite their poor “identity” as concerns the content of metal impurities, were used since their mean molar masses covered a relatively broad range of 0.43 to 1.3 MDa (cf. Table 1).

Under the conditions used in our studies (neutral pH), the HA macromolecules are present in a highly ionized state: the pK_a_ value for the D-glucuronic acid residues is 3.12 (Park and Chakrabarti, 1978). The D-glucuronic structural units of the HA polyanion form salts with transition metal counter-cations. As formerly reported (Nagy *et al*., 1998), HA is able to weakly bind cupric ions (the binding constant being 3.0×10^3^ L/mol (Figueroa *et al*., 1977). Generally, the transition metal counter-cations form coordinate complex compounds, in which the metal cation can be fixed intra- or intermolecularly simply via the carboxyl groups of HA (Nagy *et al*., 1998; Pirc *et al*., 2004). However, especially for the copper-hyaluronate coordinates, it has been suggested that in the metal ion “binding” site electrons of the nearest *N*-acetyl group from the same HA molecule might be involved along with the COO^−^ group (Magnani *et al*., 1999).

The ·OH radical, which redox potential (·OH/H_2_O) equals +2.31 V at pH 7, is classified as the most efficient initiator of the radical oxidation/degradation accepting an H· radical from the macromolecule of hyaluronan. The formation of ·OH radicals can occur in several manners, while by far the most important *in vivo* mechanism is mediated by hydrogen peroxide. Under aerobic conditions, its generation proceeds according to reactions **1** and **2**, as well as via those depicted in Scheme 1. Thus, on addition of a high excess of AA (100 μM), trace amounts (submicro/micro) of Fe and/or Cu ions present in the HA samples undergo multiple redox cycles generating a flux of ·OH radicals. A “lag-phase” registered during the experiments represented in Figure 2 (left panel) means that a given amount of initiating ·OH radicals must be generated right at the beginning. An excess amount of either Fe or Cu ions added to the system comprising hyaluronan and ascorbate yields a much higher flux of ·OH radicals (compare the results in Figs. 3 and 4 with those shown in Figure 2, left panel). The greater the metal content, the shorter or even absent is the lag-phase. Moreover, in the case of assessing the effect of CuCl_2_ added to the system (cf. Figure 4, right panel), a really dramatic decline in the η *vs*. time dependence can be observed for all HA sample solutions investigated.

The pro-oxidative effect of both biogenic transition metals, i.e. Fe and Cu, evidenced in the presented study corroborates the observations already published by several authors studying HA degradation caused by ascorbate alone or in combination with Fe and/or Cu ions (Khan and Martell, 1967; Wong *et al*., 1981; Matsumura and Pigman, 1965; Niedermeier *et al*., 1967a; 1967b; Swann, 1967; Halliwell and Foyer, 1976; Harris and Pigman, 1976; Samuni *et al*., 1983; Buettner and Jurkiewicz, 1996). However, the study protocol including the assays of the effect of another biogenic transition metal, manganous ions, may to our knowledge be classified as pioneering. When added into the system comprising hyaluronan macromolecules, the reductant – ascorbate, and the pro-oxidatively acting Fe and/or Cu ions, Mn(II) ions demonstrate a significant antioxidative effect. The high-molar-mass HA samples were protected against degradation by addition of a relatively low amount of MnCl_2_ (0.5 μM; cf. Figure 2, right panel), i.e. one comparable to Mn contents in SF of healthy persons (0.42 μM on average) (Niedermeier and Griggs, 1971). On the other hand, in the case of a “massive” load of Cu(II) ions (1.0 μM), the added MnCl_2_ was also effective (cf. Figure 5, both panels), yet it should be pointed out that not even the highest concentration applied (30 μM) was able to prevent the oxidative damage of HA macromolecules.

The changes of the chemical structure of the HA chain occurring during the metal ions-catalyzed ascorbate auto-oxidation with participation of manganous salts leading to the formation of an increased fraction of hyaluronan hydroperoxides (and/or of AOH-type derivatives) according to the reactions **10** and **11**

(10) $$\text{AO}{\text{O}}^{\cdot }\text{+ Mn(II)+}{\text{H}}^{\text{+}}\text{→AOOH + Mn(III)}$$

(11) $$\text{A}{\text{O}}^{\cdot }\text{+ Mn(II)+}{\text{H}}^{\text{+}}\text{→AOH + Mn(III)}$$

analogously to reaction **9** suggested by Coassin *et al*. (1992) and Sziraki *et al*. (1998; 1999) have yet to be proven. The hyaluronan hydroperoxides produced in this way should, however, be decomposed by the action of the two transition metal cations in lower oxidation states − Cu(I) and Fe(II).

For detection of such AOOH and/or AOH type derivatives, the non-isothermal chemiluminometry and MALDI-TOF mass spectrometry could be proposed as relevant analytical methods (Šoltés *et al*., 2006; 2007; Rychlý *et al*., 2006).

## Concluding Remarks

The structure of the electronic orbitals of iron – 1s^2^2s^2^2p^6^3s^2^3p^6^3d^6^4s^2^ – and its high redox potential value – Fe(III)/Fe(II) equaling +0.48 V at pH 7 (Koppenol, 1994) – predetermine iron as one of the major participants in the production (and metabolism) of free radicals in biological systems. While Fe(III) at physiological pH values precipitates as oxyhydroxide aggregates, compounds containing Fe(II) are soluble, though unstable, and tend to react with oxygen to form superoxide anion radical (O_2_ ·^–^) and Fe(III). Yet the biological reductant present in the system, i.e. ascorbate (Asc^−^), restores iron's lower oxidation state according to reaction **7**. Thus, the so-called “auto-oxidation” of ascorbate is actually mediated by trace amounts of transition metals, such as iron (Buettner and Jurkiewicz, 1993). In any case, it should be pointed out that the biological consequences of the interaction of ascorbate (vitamin C) with iron are not yet fully understood. Over the past decades, the pro-oxidant properties of ascorbate have also been investigated, in addition to its better explored antioxidant role.

The copper electron configuration is 1s^2^2s^2^2p^6^3s^2^3p^6^3d^10^4s^1^ and the value of its redox potential Cu(II)/Cu(I) is +0.16 V at pH 7. The bivalent Cu(II) is paramagnetic (3d^9^) and represents the most stable oxidation state of copper. Ceruloplasmin carries copper atoms in both Cu(II) and Cu(I) states. However, it has been demonstrated that a fraction of loosely bound copper may be “liberated” under certain circumstances. Moreover, reactive oxygen species appear to disrupt copper binding to ceruloplasmin, thereby impairing its normal protective function while releasing free copper, which in turn may promote oxidative pathology (Shukla *et al*., 2006). Since ascorbate acts as a powerful reducing agent with a redox potential of +0.282 V for the redox couple Asc·^–^/Asc^–^ at pH 7, it should reduce Cu(II) to Cu(I) and hence cuprous ions should be able to reduce molecules of dioxygen directly to H_2_O_2_.

The human body contains about 300 ppm of manganese; the recommended daily intake of this essential element is 3–9 mg. The Mn electron configuration is 1s^2^2s^2^2p^6^3s^2^3p^6^3d^5^4s^2^ and the Mn(III)/Mn(II) redox potential equals +1.5 V at pH 7. Manganese exists in several different oxidation states; within biological systems, however, the +2 valence prevails (Roth, 2006). Manganous ions are paramagnetic and thus detectable by EPR spectroscopy. The six-band EPR spectrum of Mn(II) has been detected in most natural (bio)products. A sub-ppm/ppm concentration of Mn in HA samples is an established fact, which should be taken into account at performing experiments (Prof. V. Brezová, Slovak Technical University, Bratislava, Slovakia, personal communication).

Various carbohydrate-based preparations with specifically designed precise structures and molecular parameters are currently believed to be the future effective tools/remedies applicable at treating a number of diseases (Volpi, 2006). So-called viscosupplementing injections of high-molar-mass hyaluronan directly into the osteoarthritic joints could be classified as one of such examples. A further step forward could be elimination or minimalization of the content of Fe and Cu and a potentially advantageous addition of an appropriate Mn(II) salt/complex to the injection mixtures to be administered at viscosupplementary treatment of OA.
